# A randomised controlled trial of a low-carbohydrate digitally-supported weight loss programme for type 2 diabetes

**DOI:** 10.1038/s41746-025-02116-w

**Published:** 2025-12-02

**Authors:** Elizabeth Morris, Jadine Scragg, Richard Stevens, Charlotte Albury, Paul Aveyard, Susan A. Jebb

**Affiliations:** 1 Nuffield Department of Primary Care Health Sciences, University of Oxford, Oxford, UK; 2 NIHR Oxford Biomedical Research Centre, Oxford, UK; 3 NIHR Oxford and Thames Valley Applied Research Collaboration, Oxford, UK

**Keywords:** Type 2 diabetes, Obesity, Randomized controlled trials

## Abstract

We evaluated the effectiveness of a low-carbohydrate digitally-supported weight loss programme for glycaemic control for people with type 2 diabetes (T2D) compared with usual primary care in a 12 month RCT. We individually randomised 115 people with T2D and BMI ≥27 kg/m^2^ recruited from 19 general practices in England, to receive either a 12-week low-carbohydrate programme with digital support, or usual care. There was no between-group difference in HbA1c change from baseline to 3 or 12 months (primary outcome; estimated mean difference (95% CI) –0.7 mmol/mol (–5.0 to 3.6), and –1.5 (–5.7 to 2.8), *p* = 0.80). Greater mean weight loss in the intervention group at 3 months (2.6 kg (0.6 to 4.6)) was not sustained by 12 months (–0.4 kg (–2.3 to 1.6)). While this digitally-delivered intervention was acceptable to patients, there was no evidence of a meaningful impact of the intervention on glycaemia or other cardiovascular risk factors beyond that achieved with usual care. This trial was prospectively registered at clinicaltrials.gov, NCT04916314, in June 2021.

## Introduction

Type 2 diabetes (T2D) affects more than 500 million people worldwide, with numbers continuing to rise^1^. Obesity is one of the strongest risk factors for T2D^2^; currently, 90% of adults with T2D also have overweight or obesity^3^. It is possible for people with T2D to achieve significant improvements in glycaemia, or even disease remission, if treated with intensive weight loss support^4^; however, access to suitable programmes is limited, and most patients with T2D rely on dietary advice from primary care professionals. In recent years, the National Health Service (NHS) in England has offered support to prevent and treat diabetes through referral to commercial providers offering digital support, a trend accelerated by the COVID-19 pandemic^5^. Observational analyses have shown that the change in weight is similar to in-person programmes^6^, and the digital format may increase uptake by certain patient groups^5^. However, trial evidence of effectiveness is lacking.

Dietary advice in these national programmes is based on the EatWell guidelines for good health in the general population, which recommend that most dietary energy comes from carbohydrate^7^. However, there is a growing interest from patients and practitioners in using low-carbohydrate dietary approaches^8,9^. Systematic review evidence supports them as a safe and effective option for weight loss and improvements in glycaemia in the short term, yielding greater reductions in HbA1c at 3 (–0·5%, 95% CI –0·7 to –0·2) and 6 months (–0·4%, 95% CI –0·6 to –0·1) than higher-carbohydrate diets^10^. However, many primary care teams lack time, resources, or expertise to deliver this support, and it is unclear how this approach could be delivered in practice.

We aimed to test whether a low-carbohydrate digitally supported weight-loss programme was effective at improving glycaemia, weight, and other markers of cardiometabolic risk, for people with T2D, compared with usual primary care for T2D.

## Results

**Fig. 1:**
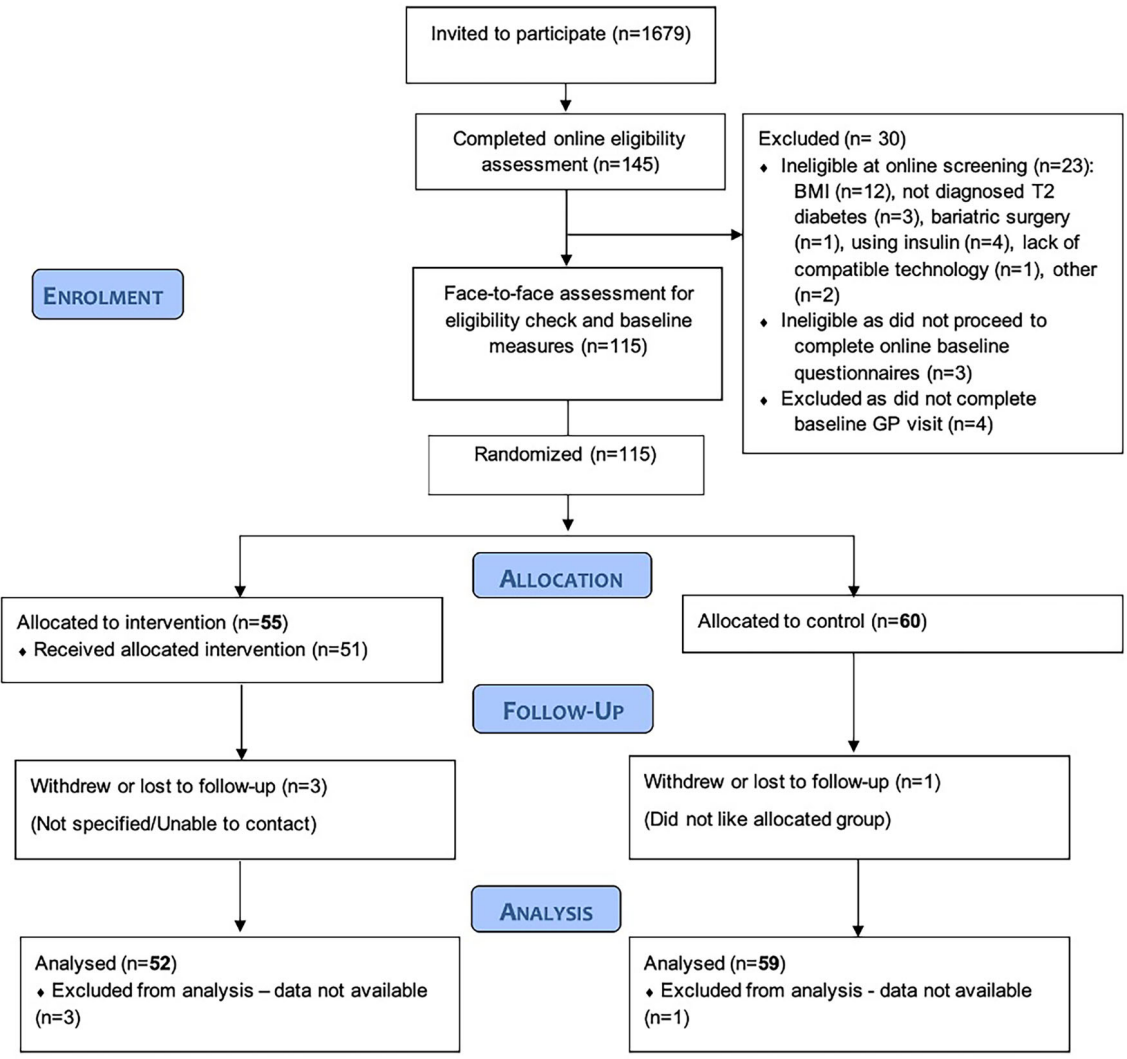
CONSORT flowchart.

Participants were recruited between 26th November 2021 and 21st July 2022. We invited 1679 patients from 20 GP practices to participate by letter, 145 (8·6%) responded by completing the online eligibility screening, of whom 115 (6·8%) from 19 practices were eligible for enrolment and were randomised (55 to the intervention, 60 to usual care). Follow-up was completed in October 2023. Follow-up data were available for 111 (96·5%) of participants at 12 months (Fig. 1); follow-up data for the primary outcome were available for 107 (93%) and 110 (95·7%) participants at 3 and 12 months, respectively.

At baseline, the average age was 58.7 years (SD 9·2), 55% were women and 94.8% self-reported white ethnicity. Mean body mass index (BMI) was 38.0 kg/m^2^ (SD 6·7), and HbA1c was 54.5 mmol/mol (SD 12·0). The average duration of T2D was 2.5 years (SD 2·1), and the average baseline PAID score of 36.6 (SD 21·4) indicated moderate diabetes distress (Table 1).

**Table 1:** Baseline characteristics of the intention-to-treat population: participants assigned to the intervention programme (*n* = 55) or usual care (*n* = 60)

	Intervention (*n* = 55)	Control (*n* = 60)	All (*n* = 115)
Age, years	60.1 (9.6)	57.3 (8.8)	58.7 (9.2)
Gender^a^			
Female	31 (56)	32 (53)	63 (55)
Male	23 (42)	27 (45)	50 (43)
Other or prefer not to say	1 (2)	1 (2)	2 (2)
Duration of diabetes, years	2.5 (2.3)	2.6 (1.8)	2.5 (2.1)
Weight, kg	104.9 (23.5)	110.0 (19.2)	107.5 (21.4)
Height, m	1.68 (0.09)	1.69 (0.09)	1.68 (0.09)
BMI, kg/m^2^	37.2 (6.9)	38.8 (6.5)	38.0 (6.7)
HbA1c, mmol/mol	51.3 (10.2)	57.4 (12.9)	54.5 (12.0)
Systolic blood pressure, mmHg	131.7 (13.0)	133.6 (14.6)	132.7 (13.8)
Diastolic blood pressure, mmHg	79.0 (9.4)	81.9 (10.0)	80.5 (9.8)
Number of medications taken for diabetes	0.9 (0.6)	1.1 (0.9)	1.0 (0.8)
Number of participants taking diabetes medications^a^	40 (72.7)	46 (76.7)	86 (74.8)
Number of participants taking antihypertensives^a^	27 (49.1)	30 (50)	57 (50.0)
Number of participants taking lipid lowering medications^a^	36 (65.5)	29 (48.3)	65 (56.5)
Ethnicity^a^			
White	51 (92.7)	58 (96.7)	109 (94.8)
Asian	3 (5.5)	1 (1.7)	4 (3.5)
Black, mixed or other ethnicity	1 (1.8)	1 (1.7)	2 (1.7)
Education^a^			
Higher education	16 (29.0)	25 (41.7)	41 (35.7)
Secondary education	31 (56.4)	24 (40.0)	55 (47.8)
Technical qualifications	4 (7.3)	4 (6.7)	8 (7.0)
No formal qualifications or prefer not to say	5 (9.1)	6 (10)	11 (9.6)
Lipid profile			
Total cholesterol, mmol/L	4.5 (1.3)	4.5 (1.0)	4.5 (1.1)
Triglycerides, mmol/L (*n* = 107)	2.3 (1.1)	2.5 (1.5)	2.4 (1.3)
HDL cholesterol, mmol/L (*n* = 110)	1.2 (0.4)	1.1 (0.3)	1.2 (0.3)
LDL(non-HDL) cholesterol, mmol/L (*n* = 99)	2.5 (1.2)	2.4 (0.9)	2.5 (1.0)
Total chol:HDL ratio (*n* = 110)	4.0 (1.3)	4.2 (1.3)	4.1 (1.3)
Liver function tests			
ALT, IU/L (*n* = 115)	31.4 (17.9)	36.1 (22.5)	33.9 (20.5)
ALP, IU/L (*n* = 110)	85.5 (25.7)	90.6 (23.9)	88.1 (24.8)
Albumin, g/L (*n* = 114)	42.8 (3.4)	42.4 (3.6)	42.6 (3.5)
PAID score	34.9 (23.0)	38.2 (19.9), 0–72.5	36.6 (21.4), 0–82.5
EQ5D-5L index score	0.689 (0.290)	0.738 (0.234)	0.714 (0.262)
EQ5D-5L VAS score	61.3 (23.0)	57.7 (18.5)	59.4 (20.7)

Values are mean (standard deviation) unless stated otherwise.

*BMI* body mass index, *HbA1c* glycosylated haemoglobin A1C, *HDL* high density lipoprotein, *LDL* low density lipoprotein, *ALT* alanine aminotransferase, *ALP* alkaline phosphatase, *PAID* problem areas in diabetes, *EQ5D-5L* EuroQuol Quality of Life assessment tool, 5D-5L version.

^a^ Number of participants (%).

### Primary outcomes

There was no statistically or clinically significant between-group difference in HbA1c change from baseline to 3- or 12-months (Table 2; *p* = 0·80). This result was consistent across pre-specified sensitivity analyses (Supplementary Table 2), and in the as-treated analysis, where 51 of 55 participants commenced the assigned intervention (Supplementary Table 1). In a post hoc sensitivity analysis, we compared multiple imputation to reference-based imputation (Supplementary Table 3) to confirm that similar results can be obtained without making the missing-at-random assumption for missing data.

**Table 2:** Primary outcome by group allocation, of participants assigned to the intervention programme (*n* = 55) or usual care (*n* = 60)

	Intervention (mean (SD))	Control (mean (SD))	Estimated effect size^a^ (95% CI)	*P* value*
Baseline HbA1c (mmol/mol)	51.3 (10.2)	57.4 (12.9)	–	
3 month HbA1c change, mmol/mol (mean, SD) (*n* = 107)	–2.3 (7.9)	–2.4 (8.5)	–0.7 (–5.0 to 3.6)	0.80
12 month HbA1c change, mmol/mol (mean, SD) (*n* = 110)	0.4 (11.9)	–1.0 (15.8)	–1.5 (–5.7 to 2.8)	

*HbA1c* glycosylated haemoglobin A1C.

^*^Omnibus likelihood ratio test: *p* = 0.80, indicating insufficient evidence to reject the null hypothesis that there is no significant change in HbA1c at 3 or 12 months.

^a^ Adjusted for baseline value and practice; positive numbers indicate greater reduction for the intervention group.

### Secondary outcomes

Mean weight change at 3 months was –3·6 kg (SD 3·6) in the intervention group and –0·7 kg (SD 5·9) in the control group, (adjusted difference 2·6 kg with 95% confidence interval 0·6 to 4·6). This significant between group difference in mean weight change had attenuated by 12 months (–0·4 kg (–2·3 to 1·6)) (Table 3). A third (18 of 55, 32·7%) of participants in the intervention group had lost ≥5% of their body weight at 3 months (compared to 5 of 60 participants (8·3%) in the control group); by 12 months, ≥5% body weight loss was 25·5% (14 of 55 participants) in the intervention group and 21·7% (13 of 60) in the control. Only 7% of all participants (4 intervention, 4 control group) achieved a ≥10% body weight loss by 12 months (Supplementary Table 4).

**Table 3:** Secondary outcomes by group allocation, of participants assigned to the intervention programme (*n* = 55) or usual care (*n* = 60)

	Intervention	Control	Estimated effect size (95% CI)	*P* value*
**Weight (kg)**				
Baseline weight (*n* = 115)	104.9 (23.5)	110.0 (19.2)		
3 month weight change (*n* = 108) (mean, SD)	–3.6 (3.6)	–0.7 (5.9)	2.6 (0.6 to 4.6)	0.0069*
12 month weight change (*n* = 109) (mean, SD)	–2.4 (5.3)	–2.7 (5.9)	–0.4 (–2.3 to 1.6)	
**Systolic BP (mmHg)**				
Baseline (*n* = 115)	131.7 (13.0)	133.6 (14.6)		
3 month change (n = 107) (mean, SD)	–2.1 (12.3)	–2.9 (13.0)	–0.8 (–6.0 to 4.4)	0.92
12 month change (*n* = 105) (mean, SD)	–1.6 (14.2)	–0.9 (15.2)	0.2 (–5.0 to 5.4)	
**Diastolic BP (mmHg)**				
Baseline (*n* = 115)	79.0 (9.4)	81.9 (10.0)		
3 month change (*n* = 107) (mean, SD)	–2.1 (9.1)	–3.2 (8.0)	–1.1 (–4.8 to 2.6)	0.35
12 month change (*n* = 105) (mean, SD)	–1.0 (9.2)	–2.2 (11.2)	–1.5 (–5.2 to 2.2)	
**Lipid profile**				
**Total cholesterol (mmol/L)**				
3 month change (*n* = 104) (mean, SD)	–0.01 (0.86)	–0.19 (0.85)	–0.12 (–0.45 to 0.20)	0.68
12 month change (*n* = 104) (mean, SD)	–0.21 (0.61)	–0.35 (0.98)	–0.13 (–0.46 to 0.20)	
**Triglycerides (mmol/L)**				
3 month change (*n* = 94) (mean, SD)	–0.07 (1.03)	–0.29 (1.28)	–0.19 (–0.64 to 0.26)	0.54
12 month change (*n* = 93) (mean, SD)	–0.25 (0.86)	–0.19 (1.29)	0.06 (–0.39 to 0.51)	
**HDL cholesterol (mmol/L)**				
3 month change (*n* = 96) (mean, SD)	0.03 (0.17)	0.04 (0.15)	–0.01 (–0.08 to 0.11)	0.67
12 month change (*n* = 99) (mean, SD)	0.07 (0.39)	0.04 (0.15)	–0.03 (–0.12 to 0.06)	
**LDL cholesterol (mmol/L)**				
3 month change (*n* = 81) (mean, SD)	–0.04 (0.52)	–0.10 (0.49)	–0.03 (–0.31 to 0.25)	0.97
12 month change (*n* = 91) (mean, SD)	–0.22 (0.62)	–0.25 (0.78)	–0.03 (–0.30 to 0.24)	
**Total cholesterol:HDL ratio**				
3 month change (*n* = 95) (mean, SD)	–0.11 (0.71)	–0.36 (0.87)	–0.21 (–0.53 to 0.12)	0.43
12 month change (*n* = 98) (mean, SD)	–0.35 (0.67)	–0.55 (1.09)	–0.16 (–0.48 to 0.16)	
**Liver function**				
ALT (iU/L)				
3 month change (*n* = 105) (mean, SD)	–3.3 (13.0)	–2.4 (10.9)	–0.3 (–5.5 to 4.8)	0.67
12 month change (*n* = 103) (mean, SD)	–2.1 (15.4)	–4.1 (16.6)	–2.3 (–7.5 to 3.0)	
**ALP (iU/L)**				
3 month change (*n* = 100) (mean, SD)	–2.0 (9.4)	–3.2 (12.5)	–1.0 (–7.3 to 5.2)	0.59
12 month change (*n* = 98) (mean, SD)	–0.2 (19.1)	–4.2 (16.4)	–3.3 (–9.5 to 3.1)	
**Albumin (g/dL)**				
3 month change (*n* = 105) (mean, SD)	0.4 (3.1)	0.8 (3.1)	0.4 (–0.6 to 1.4)	0.14
12 month change (*n* = 103) (mean, SD)	–0.6 (2.3)	0.6 (2.3)	1.0 (0.01 to 2.1)	
**PAID score**				
3 month change (*n* = 104) (mean, SD)	–12.3 (18.1)	–11.4 (16.2)	0.1 (–6.2 to 6.4)	0.34
12 month change (*n* = 102) (mean, SD)	–14.8 (17.2)	–10.0 (16.5)	4.2 (–2.1 to 10.5)	
**EQ5D-5L index value**				
3 month change (*n* = 104) (mean, SD)	0.059 (0.135)	0.004 (0.149)	–0.056 (–0.111 to –0.002)	0.12
12 month change (*n* = 103) (mean, SD)	0.041 (0.152)	0.001 (0.014)	–0.036 (–0.091 to 0.019)	
**EQ5D-5L VAS**				
3 month change (*n* = 104) (mean, SD)	5.2 (17.6)	4.0 (19.7)	–1.0 (–9.0 to 7.1)	0.75
12 month change (*n* = 102) (mean, SD)	4.5 (12.2)	5.9 (22.4)	2.2 (–5.9 to 10.3)	
**Diabetes medications**				
Baseline number of diabetes meds	0.9 (0.6)	1.1 (0.9)		
3 month change in number of diabetes meds (mean, SD)	0.04 (0.3)	0.07 (0.3)	0.03 (–0.09 to 0.2)	0.89
12 month change in number of diabetes meds (mean, SD)	0.09 (0.4)	0.1 (0.4)	0.009 (–0.1 to 0.1)	
**Number of participants taking antihypertensive medications**				
3 month change (*n*, %)	0 (0)	0 (0)		
12 month change (*n*, %)	–1 (1.8)	0 (0)		
**Number of participants taking lipid lowering medications**				
3 month change (*n*, %)	–1 (1.8)	3 (5)		
12 month change (*n*, %)	–2 (3.6)	5 (8.3)		

*BP* blood pressure, *HDL* high density lipoprotein, *LDL* low density lipoprotein, *ALT* alanine aminotransferase, *ALP* alkaline phosphatase, *PAID* problem areas in diabetes, *EQ5D-5L* EuroQuol Quality of Life assessment tool, 5D-5L version.

^*^As per the primary analysis, an omnibus likelihood ratio test was conducted for each continuous secondary outcome measure to test the hypothesis of a significant change in each variable at 3 or 12 months.

Four participants in the intervention group (7·3%) and 1 in the control group (1·7%) achieved diabetes remission at 12 months (*p* = 0·17). There was no statistically or clinically significant reduction in mean number of diabetes medications between groups at 3 or 12 months.

Small favourable changes in markers of cardiometabolic risk were observed in both groups at 3 and 12 months, with falls in systolic and diastolic blood pressure, total- and LDL-cholesterol and triglycerides, and increases in HDL-cholesterol, however, there were no statistically significant between group differences (Table 3).

Clinically significant reductions in reported diabetes distress were observed in both the intervention and control groups at 3 and 12 months, with no evidence this differed between groups (*p* = 0·34). Average improvements in EQ5D-5L index value scores met the threshold for minimally important observed differences^11^ in the intervention group (3 month mean (SD) change 0·059 (0·135), 12 month mean (SD) change 0·041 (0·152)) but not the control group, with no evidence of a between group difference in change in EQ5D (–0·056 (–0·111 to –0·002), –0·036 (–0·091 to 0·019) at 3- and 12-months, *p* = 0·12) (Table 3).

### Adverse events

No participants reported occurrences of severe hypoglycaemia or emergency hospital admissions related to their diabetes. Five participants reported unplanned hospital admissions in the first 3 months (*n* = 4 in control group, *n* = 1 in intervention group; *p* = 0·23); five participants reported further unplanned hospital admissions by 12 months (*n* = 5 in control group). None of these reported events were deemed by the study team to be related to the research procedures (Supplementary Table 5).

### Exploratory analyses and process measures

There were no statistically significant interactions between gender, IMD decile, or educational status at baseline and effect of the intervention on HbA1c (*p* = 0·51, 0·053 and 0·47, respectively).

Reported diet quality at baseline was consistent with that previously reported for the UK population^12^. Reported mean total energy intake at 3 and 12 months decreased similarly in intervention and control group participants. The reported proportion of total energy from carbohydrate decreased in the intervention group but not the control group at 3 months (adjusted mean difference 10% (4 to 16, *p* = 0·001) but at 12 months there was no significant difference (3% (–3 to 8, *p* = 0·34) (Supplementary Table 6).

**Fig. 2:**
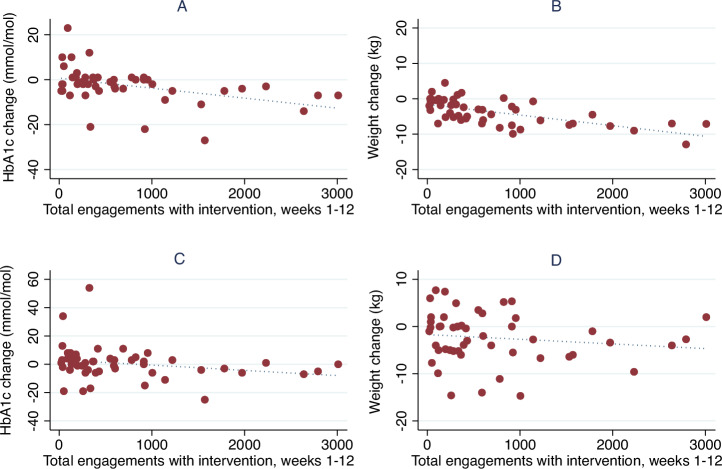
Association of changes in HbA1c and weight with total intervention engagements. **A** Association of total intervention engagements with HbA1c change at 3 months, Spearman’s correlation coefficient –0.46 (*p* = 0.0011); **B** Association of total intervention engagements with weight change at 3 months, Spearman’s correlation coefficient –0.65 (*p* ≤ 0.0001); **C** Association of total intervention engagements with HbA1c change at 12 months, Spearman’s correlation coefficient –0.31 (*p* = 0.03); **D** Association of total intervention engagements with weight change at 12 months, Spearman’s correlation coefficient –0.22 (*p* = 0.14). *Figure created using Stata v16 software (StataCorp LP, College Station, TX, USA)*.

For participants who commenced the intervention (*n* = 51), total engagement with the 12-week intervention programme was significantly associated with improvement in HbA1c at 3 (*p* = 0·0011, Fig. 2A) and 12-months (*p* = 0·03), and weight (*p* ≤ 0·0001, Fig. 2B) at 3 months, though not at 12 months (*p* = 0·14) (Table 4).

**Table 4:** Association of total number of engagements with the 12-week intervention programme, with HbA1c and weight change, in the as-treated intervention group

	Mean change (SD)	Spearman’s correlation coefficient (*p* value)
HbA1c change at 3 months (mmol/mol) (*n* = 47)	–2.6 (8.2)	–0.46 (*p* = 0.0011)
HbA1c change at 12 months (mmol/mol) (*n* = 49)	0.5 (12.2)	–0.31 (*p* = 0.03)
Weight change at 3 months (kg) (*n* = 48)	–3.8 (3.6)	–0.65 (*p* ≤ 0.0001)
Weight change at 12 months (kg) (*n* = 49)	–2.4 (5.4)	–0.22 (0.14)

### Qualitative findings

The characteristics of the 13 participants from the intervention group who participated in telephone interviews (with an average duration of 40 min) are described in Supplementary Table 7. Responses were grouped around four high-level categories: (1) Perceptions of the intervention programme. (2) Perceptions and reported experiences of low-carbohydrate dietary advice. (3) Reported experience of the digital mode of intervention delivery. (4) Facilitators of initial and ongoing engagement. Categories and data relating to perceptions and experiences of the intervention programme are presented in Supplementary Table 8 and summarised here.

Participants took part in the study to get support for weight loss, control their diabetes, and stop or avoid medication for diabetes. They sought to improve their health, and were motivated by recent illnesses. Recruitment took place in the latter half of the COVID-19 pandemic. Many participants reported that they had gained weight, had been ill, and felt they had insufficient help to manage their diabetes and were seeking connection with others. Many participants reported they were also motivated to help others by participating in research.

#### Catergory 1: perceptions of the intervention programme

Most participants reported that they found the intervention programme generally acceptable. While some expressed significant enthusiasm or reservations about the programme, many did not hold strong views. Participants reported positive impacts from the programme, including weight loss, improved glycaemia or diabetes remission, and improved emotional, physical and psychological wellbeing. However, definitions of “success” varied widely both in terms of weight loss (*“I’ve lost over a stone in weight”, interview(i)2, “I would say a decent amount of weight is anything over a kilo for me”, i6)* and other outcomes (*“I actually have been able to sleep better” i13*, “*it’s helped me think about my mental wellbeing. and being able to cope with it” i13*).

Positive or neutral perceptions of the intervention programme were reported by most participants who described it primarily as **“not a diet” but a holistic “toolkit” encouraging healthy habit formation** following sensible, simple strategies designed to be **sustainable** longer-term. Around half the participants perceived the programme to be just “healthy eating” and did not articulate low-carbohydrate eating as a key feature. They described dietary and behavioural strategies such as adoption of simple substitutes, and reducing portion sizes *(“they said you’re not really dieting, you’re more changing your habits” i9)*.

The **simple advice** and lack of emphasis on weight loss was welcomed (*“it was better because it wasn’t as strict” i7, “I’m not worried that I’m not losing weight” i11)* and many felt positive about the **flexibility** *(“There was no pressure as such, so you didn’t feel like you were a bad person for not doing something” i13)*. However, some recognised that they had not changed much in their diet and were more sceptical about this approach, and were **left wanting more** *(“In the blurb it says we don’t tell you what to eat, but we can tell you why you’re eating things well…I just like a little bit more” i8; “Not much has changed really… you stick to what you know” i6*).

#### Category 2: perceptions and reported experiences of low-carbohydrate dietary advice

Some participants did identify that a low-carbohydrate eating plan was a key principle of the programme; fewer than half of participants volunteered this spontaneously, though more described it when discussing the diet specifically. There were two main sub-categories of responses to the low-carbohydrate component: Some felt empowered by understanding how this would improve their diabetes and embraced the simplicity of the message, “**it’s a simple message to cut the carbs**”. They understood this as giving flexibility to their diet *(“It was like a lightbulb moment for me, that. you can eat really pretty much what you like, but you just cut the carbs” i5)*. Some described a **struggle** with preconceptions about “extreme” diets, the lack of palatability of the diet, and that it did not fit with their personal or cultural dietary patterns *(“I just find the food unappetizing and uninteresting” i8; “I think the content is quite important because I’m also a vegetarian, so I don’t eat meat or fish. So I find it really difficult*” i7).

#### Category 3: reported experiences of the digital mode of intervention delivery

**Receiving the support digitally** was acceptable for most participants, but very few saw this as a specific advantage *(“I found it perfectly adequate and acceptable, because I’ve never seen it in any other way” i8)*. Positive elements of the digital offering included its novelty, convenience, accessibility, and flexibility to pick and choose parts of the programme that participants felt would work for them.

Participants perceived a need to be “**tech savvy**” to engage with the digital format, which some reported was a barrier for them. *(“The only difficulty that I, personally (found), was the tech… The tech was difficult for me” i4)*. **Challenges with the technology** caused frustration and undermined motivation to engage.

While **automation** of activity tracking and self-monitoring data provided positive motivation and feedback for some participants, others felt this detracted from their engagement (removing the need to actively interact with the app). Some described **remote support** felt both practically and psychological distanced, needing to initiate contacts in order to receive help and advice was burdensome, and they wanted a personal connection.

Some participants raised concerns about **digital security** and privacy of their personal data *(“I haven’t done my steps because that’s like a map of my steps, so I haven’t done the tracking” i13)*. Others reported experiences consistent with **digital fatigue,** *(“I find (it) boring and tedious. The hour after hour tapping away at a screen” i4*).

Some participants appreciated the peer support groups –particularly if they felt they identified with ‘**people like me’**, similar in age, demographics, or working situation, when they felt encouraged to engage with the group and believed that it would be helpful *(“They were on the same mission as you to lose weight, to eat healthily. I suppose we were looking for people with the same goals” i10)*. However, the opposite was true for those who did not identify with the demographics or experiences of others in the peer support group, where this formed a barrier to their engagement *(“the group chat… different people have different method, different personalities…I didn’t think that they would interest me in the chat or that that would really help me” i12*).

#### Category 4: facilitators of initial and ongoing engagement

The remote support and interactions through the app were commonly highlighted as helpful. Alongside use of effective **behavioural strategies** such as self-monitoring and goal setting, positive reinforcement of seeing results, and enthusiasm for the **educational components** of the programme (*“quite regularly when I read the articles I thought, ‘That is something I did not know,’ and I never would have thought of it being the case” i4*), contact with the **coach** and encouragement from **peer support** were described as positive facilitators in particular for building motivation, confidence, knowledge, and for some an element of personalisation of the programme *(“I think one of the biggest things I found enjoyable was working with the life coach. she was excellent at coming back to me and that gave me the confidence to carry on” i1*).

## Discussion

In this randomised trial comparing a low-carbohydrate digitally supported weight loss programme with usual primary care for people with T2D, we found no evidence of a meaningful difference in HbA1c at 3 or 12 months, and no evidence of a sustained impact of the intervention on body weight. There was a small temporary reduction in the proportion of energy from carbohydrate in the intervention group relative to control. At 12 months, both groups had achieved clinically relevant average reductions in weight and improvements in quality of life.

Data from observational case series of people with T2D receiving low-carbohydrate dietary advice from motivated practitioners have reported diabetes remission and reductions in prescriptions of diabetes medications. However, systematic reviews of randomised studies of low-carbohydrate programmes in T2D have consistently reported only short-term improvements in weight and HbA1c, and no sustained difference in effect between lower and higher carbohydrate diets in the longer term (≥12 months)^10,13^. Our findings are in keeping with reviews of reviews of existing literature, with short-term benefits on body weight at 3 months but no sustained added benefit of referral to this programme on weight or HbA1c over and above usual care at 1 year^13,14^.

The Scientific Advisory Committee on Nutrition reported that many of the trials of low-carbohydrate diets did not report the change in carbohydrate intake and those that did, frequently found only small or no difference in carbohydrate intake between groups, partly due to poor adherence to the prescribed diet, and partly due to contamination of the control group. Here, the intervention programme aimed to achieve a dietary pattern with 20–30% total energy from carbohydrate, however, relatively few participants achieved this. In our trial, both groups reduced their energy intake by a similar amount, consistent with the similar changes we measured in body weight, but only the intervention group reduced the proportion of energy from carbohydrate, leading to a significant difference between groups at 3 months, though this was small and not sustained and the proportion of carbohydrate in the diet of both groups at 1 year was similar to that at baseline. The qualitative findings suggest that the “low-carbohydrate” message of this programme was often not effectively communicated or implemented, and the aim to encourage sustainable changes was not met. This is likely to explain why the improvements in weight and glycaemia that have been seen in other low-carbohydrate and behavioural intervention programmes for people with T2D in primary care^9^ were not seen here.

We observed significant weight loss in the control group at 12 months, suggesting that the intervention brought forward weight loss that would have been achieved anyway. Weight loss in people motivated join a trial offering support to lose weight is common. This may be a Hawthorne effect, where participation in a study and awareness of being monitored may contribute to weight loss efforts^15^. A systematic review of weight loss in control groups in trials reported that mean weight loss was about 1 kg at 3 months (95% prediction interval +1·3 to –3·3 kg) and 0·8 kg at 12 months (95% PI + 1·5 kg to –2·5 kg)^16^ while a more recent review (though without 95%PIs) reported lower mean weight losses still^17^. As such, weight loss in the control group of our trial is higher than expected. There are several possible explanations for this finding. It is possible that people with overweight living with T2D may have a stronger motivation to lose weight than those without co-morbidities. It may also have been impacted by the COVID-19 pandemic. In England, a new obesity strategy was launched in July 2020, with enhanced provision of weight management programmes and campaigns to encourage healthy lifestyles to reduce the risks of severe COVID outcomes and wider ill-health, both for personal benefit and to reduce the pressures on the NHS^18^. In our qualitative study, many participants explained that they were specifically motivated to join the study to lose weight. While data on weight loss referrals or efforts undertaken by individuals in the control group were not available for analysis, many participants opportunistically described to the study team their motivations for weight loss and strategies they had employed as individuals when disappointed with their control group allocation.

This was a pragmatic trial using an existing intervention in widespread use. We sought to recruit a population representative of those living with T2D, in particular, achieving representation across all income and educational attainment groups, reflecting the English population with T2D^19^. We recruited a population with diverse glycaemic and cardiometabolic risk factor control. However, despite efforts to achieve representative recruitment, participants of White ethnicity, and female sex, were over-represented compared to regional averages for people with T2D^19^. This meant we were unable to assess for subgroup effect of the intervention by ethnicity, due to the small sample size, and the effectiveness among people from non-White ethnic groups is unknown. We achieved full recruitment and high follow-up, providing robust evidence on the true effectiveness of this programme. Working with primary care teams as recruitment sites contributed to the high follow-up rates and completeness of data, drawing on the relationship between people with type 2 diabetes under long-term follow up and the healthcare professionals providing this, the convenience to participants of attending their local practice for study visits, and the ability to extract relevant outcome data from routine health encounters (with consent) where a participant was unable to re-attend for a study visit—with the study designed to focus on clinically and patient-reported relevant outcomes. Given the pragmatic study design, a standardised weighing scale was not used for all participants, and the use of different devices could have influenced results; however, the randomised nature of the trial should minimise the risk of systematic bias to either under- or over-report weight change between groups being introduced by the use of different digital weighing scales at follow-up appointments.

We note that the HbA1c in the intervention group was lower than that of the control group at baseline; while this was due to chance, given the nature of randomisation of individuals in trials, it could contribute to making it relatively more challenging for this group to achieve clinically significant reduction in HbA1c, and statistically less likely to detect a significant effect if one were present. Similarly, in line with the relatively short average duration of diabetes in recruited participants (mean (SD) 2.5 years (2.1)), average number of diabetes medications at baseline was relatively low (mean (SD) 0.9 (0.6)), again making it less likely that a significant change would be observed in a study of this size.

Adoption of a low-carbohydrate diet was limited. The company that made this programme make continuous changes to their programme in response to feedback and outcomes. At the time of our study, and in the context of the Covid19 pandemic, the original low-carbohydrate focus was reduced to focus more on psychological support, resilience, and general well-being. This is evident in the qualitative interviews where participants expressed only limited awareness of the low-carbohydrate component or other specific dietary restriction. The macroeconomic impact of the pandemic meant that health coach turnover was higher than usual, potentially affecting the continuity and effectiveness of coaching at this time.

Data completion rates were lower for self-reported 24 h dietary recall data (60–81% across the three time points) than other study outcomes, which is in keeping with previously published data^20^; however reported diet quality at baseline was consistent with that previously reported for the UK population which provides reassurance as to the representativeness of the data, and there was no marked difference in reporting rates between groups^12^. To avoid potential bias against data collection in patients with demographics which have been shown to be associated with difficulty self-completing 24-h dietary recalls (such as older age or lower educational experience^21^), a range of measures were implemented including offering to complete these over the phone with a member of the study team (“interviewer-administered”) and support for practice staff to offer assistance with completion at study visits. The dietary data does show clear evidence of under-reporting in both groups, relative to estimated energy expenditure^22^, that was evident at every timepoint, as is a well-recognised phenomenon with dietary recall data^23^; but expressing the carbohydrate intake as a proportion of total energy provides evidence that there was a difference between groups in carbohydrate intake, as intended, in the short-term. However, the magnitude of change was smaller than planned and not sustained. We did not interview participants randomised to the usual care group and so we are unable to comment on any perceived value of participation in the trial, and to know if this stimulated personal weight-loss endeavours that may help to explain the larger than expected weight-loss in this group.

Previous observational data have suggested that digitally supported interventions may be effective with reported weight loss of 2 to 3 kg^5^^,^^24^. Changes reported within the intervention group in this trial were similar, though the usual care arm experienced a greater weight loss than anticipated reducing the differences between groups. The confidence intervals were precise enough to be confident that this intervention did not achieve a moderate benefit to glycaemic control or weight compared to usual care at 12 months. Nonetheless, both groups lost weight and experienced improved health outcomes. Qualitative findings included improved wellbeing beyond weight loss in the intervention group, suggesting that people valued the support they received.

Observational analysis of data from national programmes can provide valuable insights into uptake, adherence and differential outcomes across a range of providers^25^. However, this randomised controlled trial highlights the limitations of observational evidence in understanding the effectiveness of programmes and also suggests a need for ongoing monitoring of the fidelity of programme delivery, recognising that they evolve over time in response to circumstances and participant feedback.

In conclusion, there was no evidence that an intervention, planned as a low-carbohydrate, digitally-supported weight loss programme for people with overweight and T2D led to greater weight loss than achieved by people motivated to join the trial but who received usual health care. There was some evidence that the intervention was not delivered as planned and the reduction in carbohydrate intake was small and transient. The digital support was acceptable to participants and some perceived benefits other than weight loss. Further research is needed to identify if a low-carbohydrate intervention can be successfully implemented at scale in routine care and if this can improve glycaemic control.

## Methods

### Study design

This study was an individually randomised controlled trial in which participants with T2D were allocated to one of two trial arms: a 12-week behavioural programme with education and remote support provided via an app (intervention) including advice to reduce carbohydrate as part of a weight loss programme or to continue with their usual NHS diabetes care at their GP practice (control). It ran from August 2021 to September 2023. NHS Research Ethics approval was obtained (ref 21/WM/0101), and the study was prospectively registered at the clinicaltrials.gov registry (NCT04916314).

### Setting and participants

We recruited participants from 19 primary care practices in England. General practitioners (GPs) searched their records for adults over 40 years old with T2D and a BMI of ≥27 kg/m^2^ (≥30 kg/m^2^ if ethnicity recorded as White) and invited them by letter to participate. Major exclusion criteria were: current use of insulin, proliferative diabetic retinopathy or maculopathy (Supplementary Note). Interested persons completed online screening, eligibility, informed consent and baseline questionnaires, and attended an in-person appointment to collect baseline measures at their GP practice.

### Randomisation and masking

After completing all baseline measures, participants were individually randomised with random permuted blocks stratified by general practice to either the intervention or control groups using REDCap (Research Electronic Data Capture)^26^ inbuilt randomisation software. The randomisation sequence was generated by an independent researcher, and embedded in REDCap by an independent programmer, with allocation concealed from the study team until intervention groups were assigned. Because of the nature of the intervention, it was not possible to blind participants, clinicians, or some researchers after treatment allocation.

### Procedures

A summary of study procedures is presented in the study diagram, (Supplementary Fig. 1).

### Intervention

The intervention was a 12-week app-based programme to support people with T2D and overweight/obesity to make dietary and lifestyle changes, available through various NHS pathways for people with obesity and/or T2D. It comprises a remote behavioural change programme with mentoring from a qualified health coach encouraging a reduced energy, low-carbohydrate diet based on a dietary pattern consisting of 20–30% total energy from carbohydrate (a range that reflects individualised advice and participant choice). It provides peer group support, structured education articles (covering diabetes education, nutritional advice, as well as more general health and wellbeing topics) in different formats (e.g. written text and video), and self-monitoring tools (for example, weight and activity tracking), and aims to encourage healthy habits that could be sustained long-term. Health coaches facilitate regular peer group conversations, as well as being available to provide personalised clinical and motivational support via private messaging. Resources were accessed via a smartphone or web-based application.

Participants randomised to the intervention group also received one additional telephone appointment with their GP or practice nurse, at the start of the 12-week intervention period, to review their current medications and assess whether medication changes were needed in view of the low-carbohydrate weight loss programme. Clinicians were provided with current guidelines on medication adjustment for people following a low-carbohydrate weight loss diet.

Participants randomised to the control group received no additional intervention and continued to receive their usual NHS diabetes care from their general practice.

### Data collection

At baseline, we collected self-reported demographic data and measured height. All other measurements were collected at baseline, 3, and 12 months, or extracted from patient electronic health records if follow-up visit data were unavailable. Body weight (kg) was measured using validated scales within GP practices. Blood pressure (BP) was measured three times, and the mean of the last two recordings calculated. Blood samples were collected to measure HbA1c, liver function and lipid profile; all blood samples were processed and analysed in NHS laboratories in line with standardised methods (no point-of-care testing was used). Validated questionnaires assessed participants self-reported quality of life (EQ5D-5L)^27^, diabetes related distress (Problem Areas in Diabetes (PAID) score)^28^, and dietary intake (via one 24 h dietary recall at each assessment timepoint, using the Intake24 dietary assessment tool), administered via an individual electronic link emailed to each participant to complete online at each timepoint^29^. Members of the research team were available to support participants via phone with data entry for these questionnaires if online data entry posed a barrier to completion. Study data were collected and managed using Research Electronic Data Capture (REDCap) tools hosted at the University of Oxford^26^.

We interviewed a purposive sample of participants from the intervention group only, (from those who consented to contact for interview, and were approached by email) aiming for diversity of age, gender, ethnicity, geographical region and index of multiple deprivation (IMD) decile, to examine their reported experience of the digitally supported intervention, after they completed the 12-week intervention period. Interviews were conducted by telephone, by EM, a female GP and clinical research fellow, and JS, a female postdoctoral researcher, both of whom held a range of experience in conducting qualitative interviews in healthcare research. The interview topic guide included open questions about their motivation for participation, and perceptions and experience of the programme and its components. It was developed collectively by the research team and patient representatives, and amended iteratively based on the interviews^30^.

### Outcomes

The co-primary outcomes of the study were change in HbA1c (mmol/mol) at 3 and 12 months. Pre-specified secondary outcomes included the number of participants achieving T2D remission (defined as HbA1c < 48 mmol/mol, off medications, at both 3 and 12 months), and changes in weight, systolic and diastolic blood pressure, lipid profile, liver function, quality of life (EQ5D-5L visual analogue score (VAS) and summary index value))^27^, diabetes related distress (PAID score)^28^, and number of diabetes, antihypertensive and lipid lowering medications.

Change in dietary intake (total energy consumption, proportion of energy from protein, fat, carbohydrate, free sugars, and total fibre consumption) was measured via the intake24 dietary assessment tool^29^ with one 24 h dietary recall submitted per participant at each of baseline, 3- and 12 months.

Process measures to examine programme engagement included previously validated metrics: measures of knowledge and education interactions (the number of articles read by participants), self-monitoring interactions (the number of times a participant recorded or viewed weight or steps readings), and support interactions (the number of messages sent or received in private or group chat channels). “Total engagement” was the sum of these interactions.

### Data analysis

Participants in observational studies of this type of intervention reduced HbA1c at 12 months by 8–10 mmol/mol^4,31^. The observed SD was 8.6 mmol/mol, with 1–2 mmol/mol (SD 6 mmol/mol) change in the control group. We aimed to test whether this was a direct effect of the intervention using a randomised controlled design. With power set at 0.95 (95%) and α set at 0·05 a difference of 8 mmol/mol between groups would require 48 participants, or with *α* = 0.025 (to allow for multiplicity using the Bonferroni method) 56 participants. In further calculations allowing for the uncertainty in estimated effect size relative to standard deviation(s), due to the novel testing of this intervention being offered in a pragmatic primary care setting and evaluated as part of an RCT (rather than observationally), we used the range of estimated effect sizes defined above (8–10 mmol/mol (SD 8.6) in the intervention arm, 1–2 mmol/mol (SD 6 mmol/mol) in the control arm), and calculated that 72 to 88 participants would be needed to maintain 95% power. In similar trials of behavioural interventions, we would expect follow-up rates of around 80%^4,32^, so we aimed to recruit 100 participants.

We followed a statistical analysis plan finalised prior to database locking, and conducted quantitative analyses using Stata v16 (StataCorp LP, College Station, TX, USA). Data are presented as mean (95% CI), and reported as intention-to-treat including data from all 115 randomised participants, unless otherwise indicated. Participants were considered lost to follow-up if there was no available follow-up data at 12 months. We used linear mixed effects regression models to assess time, group, and group × time effects for each continuous outcome (both primary and secondary), with practice included as a random intercept, and a random intercept for each participant included to account for repeated measures on the same participant. For the binary outcome of diabetes remission, we used a mixed effects logistic regression model, with remission as the dependent variable and randomised group as a fixed effect, and practice as a random effect. We used Spearman’s correlation coefficient to test the association between engagement with the intervention programme and HbA1c and weight change.

The model used for the primary outcome allowed the co-primary outcomes of HbA1c change and 3- and 12- months to be simultaneously analysed using a single model to increase efficiency in estimation compared to analysing outcomes separately. An omnibus likelihood ratio test assessed the null hypothesis that the change in HbA1c is zero at 3 months and at 12 months, against the alternate hypothesis that either or both change is non-zero. This test was carried out by comparing the primary model with interaction terms of group x time at both follow-up times, with a model containing no interaction terms between group and time. Secondary outcomes measured at both time points were analysed in the same way. In line with missing data recommendations for RCTs with longitudinal data^33^, our primary analysis fitted mixed models by likelihood methods. In sensitivity analyses to assess for any effect of missing data, we conducted prespecified baseline observation (BOCF) and last observation carried forward (LOCF) analyses, and additional post-hoc analyses comparing multiple imputation to missing not at random (MNAR) reference-based imputation (jump-to-reference) following previously described methods^34^ and implemented in Stata version 16 using the “mimix” command package. Due to limitations of this software package (regarding interaction terms, covariates and nested random effects), in this MNAR sensitivity analysis we analysed change from baseline for the primary outcome (HbA1c), and omitted the nested random effects adjustment for GP practice; variables included in these simplified models were HbA1c change, randomisation group, participant identifier, and timepoint of measurement. Results from this reference-based imputation analysis were compared to a similarly simplified multiple imputation analysis, both using 10 imputations, to investigate sensitivity to the MAR assumption of multiple imputation.

Other secondary analyses included a pre-specified “as treated” analysis for the primary outcome (HbA1c change) and secondary outcome of weight change to assess the effect of receiving the intervention as intended. We conducted exploratory subgroup analyses to assess whether treatment effects on HbA1c change differed by gender (self-reported, Male / Female), IMD decile (IMD decile of the GP practice, range 1–10, from Public Health England data^35^) or educational status (binary variable calculated from self-reported data: attended higher education (university undergraduate or postgraduate degree) vs not (GCSE, A level, vocational qualification, or other)).

We analysed qualitative data transcribed verbatim from audio-recorded interviews following a conventional content analysis approach^36^. We coded Interview data line-by-line and codes were grouped into descriptive categories and sub-categories. Analysis was led by EM and included peer discussion with JS and CA. Data management was supported by NVivo software (version 12).

## Supplementary information

Supplementary information

## Data Availability

Requests for access to data from the RESULT study should be addressed to the chief investigator. All the individual participant data collected, after deidentification (including the data dictionary) will be available on request following publication. All proposals requesting data access will need to complete a data request form with details of the research question and analysis plan. All proposals will require the approval of the investigator team before any data are released.
