# Supplementary material for: A randomised controlled trial of a low-carbohydrate digitally-supported weight loss programme for type 2 diabetes
Source: NPJ Digit Med. 2025 Dec 2;8:739. doi: 10.1038/s41746-025-02116-w (PMC12672577; doi:10.1038/s41746-025-02116-w)
Supplement: Supplementary file 1 — Supplementary information [file 41746_2025_2116_MOESM1_ESM.pdf]

Supplementary Data Appendices

Supplementary Note: Inclusion and Exclusion criteria

Inclusion Criteria

- Participant is willing and able to give informed consent for participation in the study.
- Male or Female, aged 40 years or above.
- Diagnosed with current type 2 diabetes (i.e. not in remission) in the last 6 years
- BMI of ≥27kg/m^2 (≥30kg/m^2 if ethnicity recorded as white)
- Has a smartphone or computer with internet access (and the correct operating system requirements to use the intervention programme)
- Are able to complete the eligibility and baseline assessments online
- Would like to make changes to their diet or lifestyle to improve their diabetes control, lose weight, or improve their general health

Exclusion Criteria

The participant may not enter the study if ANY of the following apply:

- Unable to understand the study materials and interventions
- Currently following a weight loss programme (defined as a structured, prescribed and monitored programme and not a self-directed weight loss attempt)
- Pregnant, breastfeeding, or planning to become pregnant during the course of the study
- History of bariatric surgery, including gastric banding
- Currently using insulin therapy
- Proliferative diabetic retinopathy, or maculopathy.
- Recent myocardial infarction or stroke (<3 months)
- Renal failure (chronic kidney disease stage 4 or 5)
- Current active treatment for cancer (other than skin cancer treated with curative intent by local treatment only)
- Their doctor does not feel they are appropriate to participate for another reason (e.g. active eating disorder diagnosis, significant psychological disturbance)

Supplementary Figure 1: Study diagram

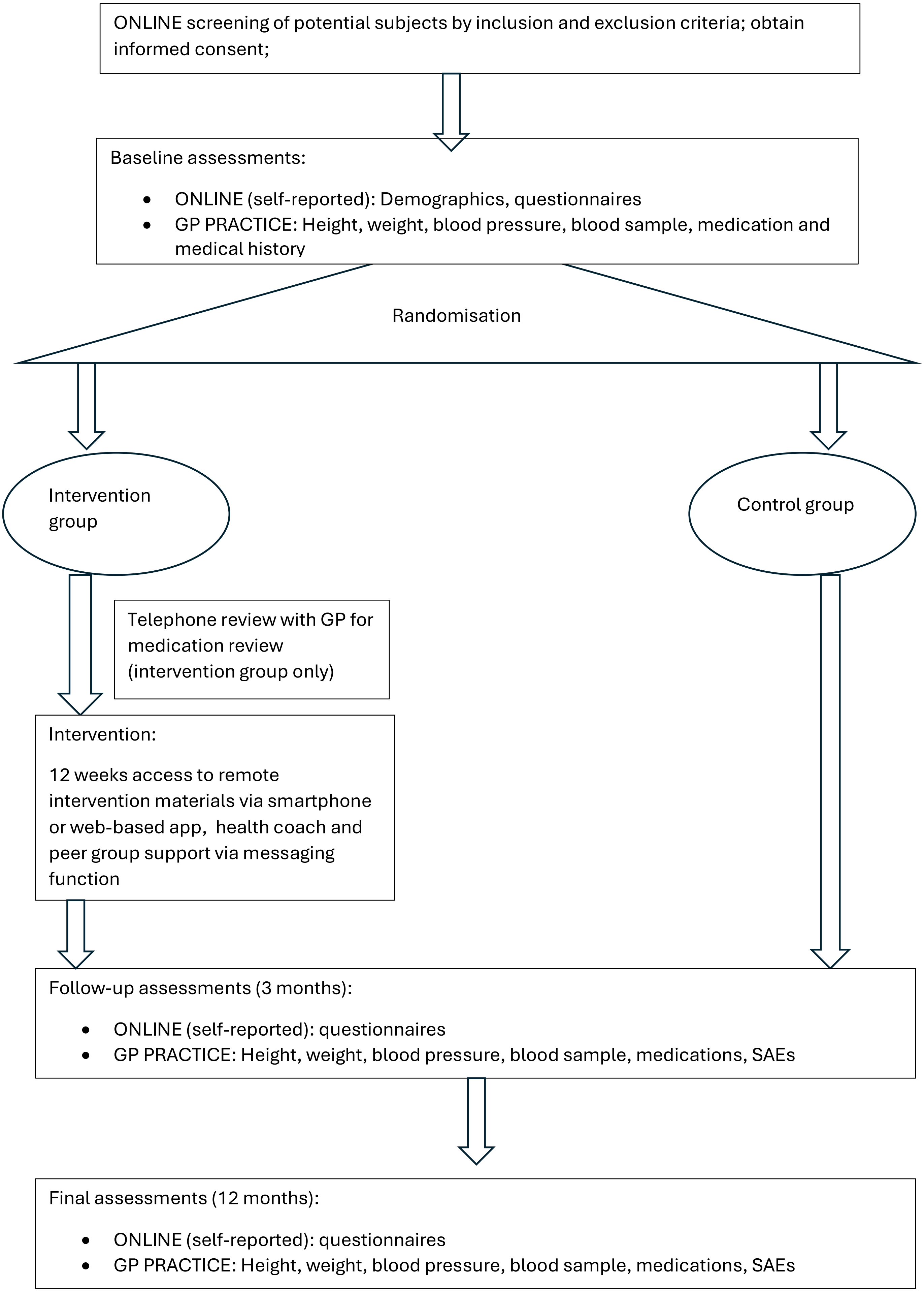

Pre-specified sensitivity analyses for the primary outcome

Supplementary Table 1: As-treated sensitivity analysis for change in HbA1c at 3 and 12 months

	Received Intervention (n=51)	Did not receive intervention (n=64)	Estimated mean difference (95% CI)	p value
Baseline HbA1c (mmol/mol) (n=115)	51.5 (10.5)	56.8 (12.7)		
3 month HbA1c change, mmol/mol (mean, SD) (n=107)	-2.6 (8.2)	-2.2 (8.3)	-0.3 (-4.6 to 4.0)	0.791
12 month HbA1c change, mmol/mol (mean, SD) (n=110)	0.5 (12.2)	-1.0 (15.5)	-1.4 (-5.7 to 2.9)	

Supplementary Table 2: Baseline Observation Carried Forward (BOCF) and Last Observation Carried Forward (LOCF) sensitivity analyses for the primary outcomes of HbA1c change at 3 and 12 months

	**Intervention (mean (SD))**	**Control (mean (SD))**	**Estimated mean difference (95% CI)**	**p value**
Baseline HbA1c (mmol/mol)	51.3 (10.2)	57.4 (12.9)		
**BOCF**				
3 month HbA1c change, mmol/mol (mean, SD) (n=115)	-2.2 (7.6)	-2.2 (8.3)	-0.04 (-4.1 to 4.1)	0.754
12 month HbA1c change, mmol/mol (mean, SD) (n=115)	0.4 (11.5)	-1.0 (15.6)	-1.4 (-5.5 to 2.3)	
**LOCF**				
3 month HbA1c change, mmol/mol (mean, SD) (n=115)	-2.2 (7.6)	-2.2 (8.3)	-0.04 (-4.1 to 4.1)	0.662

12 month HbA1c change, mmol/mol (mean, SD) (n=115)	0.6 (11.6)	-1.1 (15.6)	-1.7 (-5.8 to 2.4)	

Post-hoc sensitivity analysis for the primary outcome

Supplementary Table 3: Multiple Imputation Missing at Random (MAR) and Missing Not At Random (MNAR) reference-based sensitivity analyses for the primary outcomes of HbA1c change at 3 and 12 months

	**Estimated effect size (95%CI)**	**p value**
**MAR sensitivity analysis**		
3 month HbA1c change (mmol/mol)	-0.4 (-3.5 to 2.7)	0.79
12 month HbA1c change (mmol/mol)	-1.9 (-7.1 to 3.3)	0.47
**MNAR MI J2R sensitivity analysis**		
3 month HbA1c change (mmol/mol)	-0.3 (-3.4 to 2.7)	0.82
12 month HbA1c change (mmol/mol)	-1.8 (-7.1 to 3.4)	0.50

Supplementary Table 4: Percentage of participants achieving ≥5% or ≥10% body weight at 3 and 12 months.

	Intervention (n=55)	Control (n=60)	All (n=115)
≥5% body weight loss at 3 months	18 (32.7)	5 (8.3)	23 (20.0)
≥10% body weight loss at 3 months	1 (1.8)	2 (3.3)	3 (2.6)
≥5% body weight loss at 12 months	14 (25.5)	13 (21.7)	27 (23.5)
≥10% body weight loss at 12 months	4 (7.3)	4 (6.7)	8 (7.0)

Supplementary Table 5: Adverse events of special concern

Timepoint	PID	Intervention group	Control group
3 months	R0073	Hospital admission: chest infection	
	R0025		Hospital admission: Tic bite
	R0062		Hospital admission: Gallstone related (pre-existing condition)

	R0067		Hospital admission: Myocardial infarction
	R0103		Hospital admission: ICU admission for sepsis
12 months	R0002		Hospital admission: Abdominal pain ?gallstones
	R0010		Hospital admission: Bowel surgery
	R0018		Hospital admission: Cough syncope
	R0021		Hospital admission: Pyelonephritis
	R0062		Hospital admission: Cholecystectomy

Supplementary Table 6: Process measure analysis: diet quality

	**Overall (mean (SD))**	**Intervention (mean (SD))**	**Control (mean (SD))**	**Intervention (change in mean (SD))**	**Control (change in mean (SD))**	**Adjusted change**
**BASELINE**	*n=93*	*n=43*	*n=50*			
Total energy (TE) (kcal)	1790 (924)	1794 (961)	1786 (899)			
% TE from carbohydrate	48 (13)	49 (11)	48 (14)			
%TE from fat	34 (11)	35 (8)	34 (12)			
%TE from protein	17 (5)	17 (5)	18 (5)			
%TE from free sugars	9 (8)	10 (9)	8 (6)			
Total fibre (g)	13.3 (8.9)	12.6 (6.0)	13.8 (10.8)			
**3 MONTHS**	*n=69*	*n=32*	*n=37*	*n=28*	*n=28*	
Total energy (TE) (kcal)	1333 (678)	1332 (733)	1334 (636)	-403 (567)	-578 (928)	28 (-270 - 327), p0.852
%TE from carbohydrate	46 (14)	42 (16)	49 (12)	-7 (13)	5 (14)	10 (4 - 16), p0.001*
%TE from fat	36 (13)	38 (15)	35 (11)	2 (14)	-2 (14)	-4 (-9 – 2), p0.210
%TE from protein	18 (6)	18 (6)	18 (6)	1 (7)	1 (8)	0 (-4 – 3), p0.898
%TE from free sugars	6 (5)	5 (5)	6 (5)	-2 (9)	-2 (7)	1 (-2 – 4), p0.571
Total fibre (g)	12.2 (7.6)	12.2 (6.9)	12.2 (8.3)	-0.9 (7.5)	-4.1 (13.9)	-2 (-5 – 2), p0.322
**12 MONTHS**	*n=90*	*n=42*	*n=48*	*n=33*	*n=37*	
Total energy (TE) (kcal)	1255 (575)	1260 (553)	1251 (599)	-611 (1059)	-435 (890)	100 (-174 -374), p0.474

%TE from carbohydrate	49 (13)	48 (14)	50 (12)	-1 (12)	3 (12)	3 (-3 - 8), p0.342
%TE from fat	34 (11)	34 (11)	33 (10)	-1 (11)	-1 (12)	-1 (-6 – 4), p0.652
%TE from protein	20 (6)	20 (6)	20 (6)	3 (8)	2 (6)	-1 (-4 – 2), p0.693
%TE from free sugars	6 (7)	7 (5)	6 (9)	-3 (8)	0 (11)	1 (-2 – 4), p0.388
Total fibre (g)	11.1 (5.9)	10.7 (5.1)	11.4 (6.6)	-1.7 (7.6)	-2.5 (12.4)	0 (-3 – 2), p0.737

Supplementary Table 7: Demographics of participants who participated in qualitative interviews

Interview number	Sex		Age		Ethnicity		Region	IMD decile of practice
	M	F	<60 years	>60 years	White	Non-White		
1	X			X	X		YH	8
2	X			X	X		YH	9
3		X	X		X		YH	5
4	X			X	X		YH	9
5		X		X	X		YH	1
6	X		X		X		NW	7
7		X	x		X		TV	7
8		X		X	X		EA	10
9		X		X	X		EA	10
10		X		X	X		EA	6
11	X		X		X		EA	10
12	X		X			X	YH	1
13		x	X			x	YH	7

Abbreviations: Yorkshire & Humber (YH), North West Coast (NW), Thames Valley (TV), Eastern (EA)

Supplementary Table 8: Qualitative findings from content analysis: Categories and data relating to perceptions and experiences of the intervention programme**. (i)x denotes interview number, linked to participant demographics in table A5.**

Category	Subcategory	Example data*	Analysis
Perceptions of the intervention programme			
"Not a diet" but a holistic "toolkit"	"Not a diet" but a holistic "toolkit" for healthy actions and habit formation	"I just think, if I could describe it, it was sort of like a toolkit. There are easy things that you can do to avoid diabetes. Learning them meant that the difficult things were a lot easier"(i4)<br><br>"they said you're not really dieting, you're more changing your habits" (i9)	Most participants reported positive or neutral perceptions of the intervention programme, describing it primarily as a holistic "toolkit" encouraging healthy habit formation following sensible, simple strategies designed to be sustainable longer-term, rather than a "diet". Around half the participants perceived the diet to be just "healthy eating" and described dietary and behavioural strategies such as adoption of simple substitutes, and reducing portion sizes.<br><br>Many felt positive about the flexibility and simple messaging although some recognised that they had not changed much in their diet and were more sceptical about this approach. This flexibility of approach and perceived lack of pressure to make large changes was generally well-received and felt to be a positive feature, but appeared to contribute to lowering people's expectations of themselves and the programme in what it might help them to achieve.
	Perception as sustainable as a "marathon not a sprint"	"As they say on the plan, which was a great thing to say, it's not a sprint, it's a marathon. I found that really helpful, with your own pace, thinking about long term rather than short term goals." (i7)	
	Simple advice and simple changes can be both a positive feature or leave participants wanting more	"Not much has changed really. I've been given new ideas through the articles and the recipes and all that sort of stuff.. but you stick to what you know" (i6)<br><br>"In the blurb it says we don't tell you what to eat, but we can tell you why you're eating things well…I just like a little bit more" (i8)<br><br>"There was no pressure as such, so you didn't feel like you were a bad person for not doing something. That's a good thing because I don't respond well if somebody's trying to push something down your neck"; (i13)	
Dietary advice	"Healthy eating"	"I would just describe it as a sensible eating plan. And to be more aware of what you're eating and why you are eating it" (i7)	Some participants did identify that a low-carbohydrate diet was a key principle of the programme; fewer than half of participants volunteered this spontaneously, though more described it when discussing the diet specifically. There were two main groups of responses to the low carbohydrate component (see below)
	A low-carbohydrate diet	(see below)	
Perceptions and experiences of low-carbohydrate dietary advice			
"It's a simple message to	New information and education	"I knew about dieting, but it's something that I've struggled with, and I didn't realise the link is totally with the carbs that you eat.. People can throw recipes	Some participants felt empowered by the low-carbohydrate dietary message, by understanding how this would improve

'cut the carbs'"		at you, but this was very specific about what you could eat and what you couldn't eat, and I'd never had that before" (i5)	their diabetes – and identifying that this was novel and helpful information for them.<br>They embraced the simplicity of the message to "cut the carbs". They perceived this as giving flexibility to their diet, representing a lack of dietary restriction once they recognised all the foods they could still eat when following this approach, leaving them with freedom of choice within a set of easy to follow principles, and valued not having the additional burden of a more traditional calorie counting dietary approach.
	Freedom of choice within simple rules	"It was like a lightbulb moment for me, that.. you can eat really pretty much what you like, but you just cut the carbs" (i5)	
	Lack of restriction	"I'm not denying myself, I just adapt what I have" (i5)	
Struggles and challenges	Discord with the principles of a low-carb diet	"I think if you're fat you shouldn't cut out carbs completely. All these very extreme diets that recommend you to cut out carbs aren't coming from a position of being helpful or healthy" (i3)	*S*ome participants described struggling with the low-carbohydrate dietary advice, and several challenges that it posed. Some participants reported preconceptions about low-carb diets being "extreme" diets and perceived as unhealthy. Many identified that carbohydrates were a "problem" for them, but found it inherently too challenging to cut them out of their diet; for many people, they responded to this challenge, and the flexible ethos of the programme, by taking on some of the dietary principles and adapting them – for example, not reaching the level of carbohydrate restriction recommended, but applying the "reduced portion size" principle to this component of the dietary advice.<br><br>Other challenges included<br>the lack of palatability of the diet, and that it did not fit with their personal or cultural dietary patterns and so felt it was not well suited to them.
	Adapting the dietary principles	"It's mostly carbohydrates were, and still are, my main problem. I eat far too many of them, it's so easy to do so. But it's also a case of, you control it, you don't eliminate it" (i3)	
	Palatability and sustainability	"I have found the lack of carbohydrates, not really to my taste. I'm greedy about carbs, so that's what's causing my problem. But it's been a real difficulty sticking to the lower amounts of carbs. But I have cut down and I think that is significant in itself" (i10)<br><br>"I just find the food unappetizing and uninteresting quite a lot" (i8)	
	Cultural dietary patterns and personal relevance	"I think the content is quite important because I'm also a vegetarian, so I don't eat meat or fish. So I find it really difficult" (i7)<br><br>"There's no real reference to whether a certain type of curry or a certain amount of chapatti has what effect? So, any recipe that will be not relevant to my Asian style of cooking, I wouldn't even… it's not going to be long lasting" (i12)	
Perceptions of digital mode of delivery			
Reaction to intervention programme as a digital offering	Ambivalence	"I didn't find it off-putting that it was online" (i1)<br><br>"I found it perfectly adequate and acceptable, because I've never seen it in any other way" (i8)	Most participants found that receiving the support digitally was acceptable, but very few saw this as a specific advantage and were ambivalent in their description of this approach.
	Novelty	"I was quite excited about it because I like my apps" (i7)	Positive elements of the digital offering included its novelty, convenience,

**Positive elements of digital format**	Convenience, flexibility and accessibility	"It's convenient and wherever I go, it's always with me and when I had to go for a (social occasion) I was very mindful of what I ate, because it was on my app, and because if you are just doing it in a diary or something you could easily just ignore it and then regret what you didn't do" (i13)<br><br>"It is very, very accessible, because you can, ten minutes here, ten minutes there, you can read, as I say, your day, you can track your journal and everything" (i5)	accessibility, and flexibility to pick and choose parts of the programme that participants felt would work for them. The automation of activity tracking and self-monitoring data also provided positive motivation and feedback for some participants.
	Automated self monitoring and tracking as a motivational tool	"It not only put what your weight was, it also shows you like a graph so that it actually said whether you were doing up or down or what you were doing. And I think that helps because I think if you can see that line going gradually down, it really boosts your confidence to keep going" (i9)<br><br>"It's all visible, it's on your phone, you're being tracked, it's monitored, so it keeps your motivation quite high" (i5)	
Challenges of digital intervention programme format	Being "tech savvy" is a requirement and a potential barrier	"The only difficulty that I, personally (found), was the tech... The tech was difficult for me" (i4)<br><br>"If there are people who are not savvy with their phones and with apps, they might find it difficult.. I'm not the most tech savvy but at least I know how to work this app" (i13)	Participants perceived a need to be "tech savvy" to engage with the digital format, which some reported was a barrier for them in engaging with the programme, or perceived that it could form a barrier for others while not wanting to identify as "technically challenged" themselves - feeling they coped well despite the challenges. Where participants had experienced challenges with the technology, this caused frustration and undermined motivation to engage. Some also perceived the target demographic of the app to be different to their own – due to the digital presentation, or demographics of the coaches or others in peer support groups; where they did not identify the programme or support to be designed for "people like me", this negatively impacted their motivation to engage and perception of whether the programme offered something useful for them.<br>While the automation of activity tracking and self-monitoring data provided positive motivation and feedback for some participants, others described that this detracted from their engagement (removing the need to actively interact with the app), or raised concerns about digital security and privacy of their
	Practical and technological challenges exist and can undermine motivation to engage	"The scales were easy enough to use, the tracker was a bit more complicated. There were no help or guidance within the instruction page. It was just on how to set you up. If you press the wrong button and got into the wrong bit on the tracker, took you ages to come out and find answers" (i10)<br><br>"I really needed somebody to go, you press this and do that, and then do that, rather than having to email inquiries. It all just added to irritation for me. I was irritated at the tech. And then I sort of did master it one day, then they updated it and wiped it all, so I am furious about that" (i4)	
	The importance of identifying with "people like me" which can facilitate, or alienate in its absence	"It was the first online diet I followed, and I think the demographic is far different. It's got a very young, healthy, positive demographic, I think, of which I'm not necessarily one, you know, because I, there are lots of things about it, and I am quite old" (i8)<br><br>"the group chat… different people have different method, different personalities… I've got kids, I'm busy, so there are people who may have more time than I have…I didn't think that they	

		would interest me in the chat or that that would really help me." (i12)<br><br>"They were on the same mission as you to lose weight, to eat healthily, to improve their sleep, if that was one of their things. I suppose we were looking for people with the same goals, that's what you were trying to hope for when you joined the group, isn't it (i10)	personal data. Some participants reported experiences consistent with digital fatigue which further limited their engagement with the programme.<br><br>They also identified limitations of this format of remote support, including feeling both practically and psychological distanced from the support, the burden of needing to initiate contacts in order to receive help and advice, and missing a personal connection.
	Limitations of remote support	"In my opinion, messages doesn't bring across the personality. It doesn't bring across the person's keen to help you. just "how are you, everything going ok?" and that's it. As if somebody is just ticking the box that has sent me a message in. when somebody gives you the impression of being heard or listened, then you talk, and then somebody can give you direction and then they will find that they can make positive changes" (i12)	
	Digital fatigue	"If you work, it's quite time consuming..on the phones, well you have to read every day and you have to input every day and your weight… And just read the feedback and all the rest of it..it's quite a long time in your day and I didn't do everything" (i5)<br><br>"I chose not to do (the activities). Quite frankly, I find them boring and tedious. The hour after hour tapping away at a screen" (i4)	
	Concerns about digital security	"You're trusting them with all your information, your weight, your personal details, all of the things that you have problems with really,. You're putting it all out there" (i10)<br><br>"I haven't done my steps because that's like a map of my steps, so I haven't done the tracking" (i13)	
Facilitators of initial and ongoing engagement			
Behaviour change strategies	Goal setting and self monitoring	"They gave me a goal of I think it was 14 stone 7.. I could get under 15, I was quite happy and I did. … It was something to aim at, you know. There was no goal at all, even in my own head, I'd nothing to aim for. Well, having it like that I've actually got something to aim towards, which I felt was really good" (i9)	Participants reported using several behaviour change strategies, including goal setting (facilitated by the intervention programme format which encouraged them to do this and helped to set recommended goals), and self-monitoring which in particular gave positive reinforcement to continue with the programme when they observed positive changes and progress towards their goals.
	Positive reinforcement from observing change and progress	"Very quickly you see results and.. it just motivates you to keep doing it" (i5)<br><br>"It not only put what your weight was, it also shows you like a graph so that it actually said whether you were doing up or down or what you were doing. And I think that helps because I think if you can see that line going gradually down, it	

		really boosts your confidence to keep going" (i9)	
Educational value	Novel information and new ways of thinking about things	"The library and articles and all the resources are fantastic.. Just hints and tips really and knowledge as well. Because at the time when we first started it, I didn't really have that much knowledge about my condition" (i6)<br><br>"Quite regularly when I read the articles I thought, 'That is something I did not know,' and I never would have thought of it being the case" (i4)<br><br>"All the things that you can read, going into different things like exercise and nutrition, these were the sort of things that I'd never had access to" (i1)	Most participants expressed particular enthusiasm for the educational components of the intervention, reporting that it provided novel information or helped them to consider new ways of thinking about topics they may not have previously considered relevant to their health.
Coach and peer support interactions	Building knowledge, confidence, and motivation to continue	"I think one of the biggest things I found enjoyable was working with the life coach. The life coach that we had, very very good, plenty of information. The times when I had to contact her to clarify things she was excellent at coming back to me and that gave me the confidence to carry on because I thought this is really something" (i1)<br><br>"I think it's having somebody there and on and off they would send personal messages to you, just to check in" (i12)<br><br>"Someone that you'd could contact, with the information that was given and the encouragement that was given, and other people on the course as well, you know they put comments, I come back to them, and they were saying how good they were feeling and sending messages to each other encouraging each other" (i1)	For many participants, the remote support and interactions through the app were helpful. Contact with the coach and encouragement from peer support were described as positive facilitators in particular for building motivation, confidence, knowledge, and for some an element of personalisation of the programme.

Supplementary table 9: CONSORT 2010 checklist of information to include when reporting a randomised trial*

Section/Topic	Item No	Checklist item	Reported on page No
**Title and abstract**			
	1a	Identification as a randomised trial in the title	1
	1b	Structured summary of trial design, methods, results, and conclusions (for specific guidance see CONSORT for abstracts)	2
**Introduction**			
Background and objectives	2a	Scientific background and explanation of rationale	2
	2b	Specific objectives or hypotheses	3
**Methods**			
Trial design	3a	Description of trial design (such as parallel, factorial) including allocation ratio	11
	3b	Important changes to methods after trial commencement (such as eligibility criteria), with reasons	n/a
Participants	4a	Eligibility criteria for participants	11
	4b	Settings and locations where the data were collected	11
Interventions	5	The interventions for each group with sufficient details to allow replication, including how and when they were actually administered	12
Outcomes	6a	Completely defined pre-specified primary and secondary outcome measures, including how and when they were assessed	13
	6b	Any changes to trial outcomes after the trial commenced, with reasons	n/a
Sample size	7a	How sample size was determined	14
	7b	When applicable, explanation of any interim analyses and stopping guidelines	n/a
Randomisation:			
	8a	Method used to generate the random allocation sequence	12

Sequence generation	8b	Type of randomisation; details of any restriction (such as blocking and block size)	12
Allocation concealment mechanism	9	Mechanism used to implement the random allocation sequence (such as sequentially numbered containers), describing any steps taken to conceal the sequence until interventions were assigned	12
Implementation	10	Who generated the random allocation sequence, who enrolled participants, and who assigned participants to interventions	12
Blinding	11a	If done, who was blinded after assignment to interventions (for example, participants, care providers, those assessing outcomes) and how	12
	11b	If relevant, description of the similarity of interventions	n/a
Statistical methods	12a	Statistical methods used to compare groups for primary and secondary outcomes	14
	12b	Methods for additional analyses, such as subgroup analyses and adjusted analyses	14
**Results**			
Participant flow (a diagram is strongly recommended)	13a	For each group, the numbers of participants who were randomly assigned, received intended treatment, and were analysed for the primary outcome	P3; and fig 1
	13b	For each group, losses and exclusions after randomisation, together with reasons	Fig 1
Recruitment	14a	Dates defining the periods of recruitment and follow-up	3
	14b	Why the trial ended or was stopped	n/a
Baseline data	15	A table showing baseline demographic and clinical characteristics for each group	Table 1
Numbers analysed	16	For each group, number of participants (denominator) included in each analysis and whether the analysis was by original assigned groups	Table 2, 3
Outcomes and estimation	17a	For each primary and secondary outcome, results for each group, and the estimated effect size and its precision (such as 95% confidence interval)	Table 2, 3
	17b	For binary outcomes, presentation of both absolute and relative effect sizes is recommended	n/a
Ancillary analyses	18	Results of any other analyses performed, including subgroup analyses and adjusted analyses, distinguishing pre-specified from exploratory	P3-4, Table 3

Harms	19	All important harms or unintended effects in each group (for specific guidance see CONSORT for harms)	4
**Discussion**			
Limitations	20	Trial limitations, addressing sources of potential bias, imprecision, and, if relevant, multiplicity of analyses	9
Generalisability	21	Generalisability (external validity, applicability) of the trial findings	8
Interpretation	22	Interpretation consistent with results, balancing benefits and harms, and considering other relevant evidence	8
**Other information**			
Registration	23	Registration number and name of trial registry	P2
Protocol	24	Where the full trial protocol can be accessed, if available	Clinical trial registry clinicaltrials.gov
Funding	25	Sources of funding and other support (such as supply of drugs), role of funders	16

Citation: Schulz KF, Altman DG, Moher D, for the CONSORT Group. CONSORT 2010 Statement: updated guidelines for reporting parallel group randomised trials. BMC Medicine. 2010;8:18.
© 2010 Schulz et al. This is an Open Access article distributed under the terms of the Creative Commons Attribution License (http://creativecommons.org/licenses/by/2.0), which permits unrestricted use, distribution, and reproduction in any medium, provided the original work is properly cited.

*We strongly recommend reading this statement in conjunction with the CONSORT 2010 Explanation and Elaboration for important clarifications on all the items. If relevant, we also recommend reading CONSORT extensions for cluster randomised trials, non-inferiority and equivalence trials, non-pharmacological treatments, herbal interventions, and pragmatic trials. Additional extensions are forthcoming: for those and for up-to-date references relevant to this checklist, see www.consort-statement.org.

Supplementary Table 10: COREQ checklist

**Domain 1: research team and reflexivity**		
**Personal characteristics**		
1. Interviewer/facilitator	Which author(s) conducted the interview or focus group?	Two authors (EM and JS) conducted the interviews
2. Credentials	What were the researcher's credentials? (e.g. PhD, MD)	JS has a PhD, EM has a BMBCH (medical doctorate)
3. Occupation	What was their occupation at the time of the study?	EM and JS were research fellows
4. Gender	Was the researcher male or female?	EM and JS are female
5. Experience and training	What experience or training did the researcher have?	EM and JS had both undertaken training in qualitative research methodologies and had previous experience of this research method
**Relationship with participants**		
6. Relationship established	Was a relationship established prior to study commencement?	No prior relationship was established between the researchers and participants

7. Participant knowledge of the interviewer	What did the participants know about the researcher? (e.g. personal goals, reasons for doing the research)	Participants knew where the researchers worked and the purpose of the research
8. Interviewer characteristics	What characteristics were reported about the interviewer/facilitator? (e.g. bias, assumptions, reasons and interests in the research topic)	The researchers both had an interest in the research subject. EM has worked with patients with type 2 diabetes in primary care, and in research of dietary interventions for people with T2D.
**Domain 2: study design**		
**Theoretical framework**		
9. Methodological orientation and theory	What methodological orientation was stated to underpin the study? (e.g. grounded theory, discourse analysis, ethnography, phenomenology, content analysis)	We analysed qualitative data transcribed verbatim from audio-recorded interviews following a conventional content analysis approach
**Participant selection**		
10. Sampling	How were participants selected? (e.g. purposive, convenience, consecutive, snowball)	We interviewed a purposive sample of participants from the intervention group only, (from those who consented to contact for interview, and were approached by email) aiming for diversity of age, gender, ethnicity, geographical region and index of multiple deprivation (IMD) decile,
11. Method of approach	How were participants approached? (e.g. face to face, telephone, mail, e-mail)	Participants were approached by email

12. Sample size	How many participants were in the study?	13 participants
13. Non-participation	How many people refused to participate or dropped out? Reasons?	Numbers of refusals were not recorded; no participants dropped out once interviewed
**Setting**		
14. Setting of data collection	Where was the data collected? (e.g. home, clinic, workplace)	Interviews were conducted and recorded by telephone so participants could choose to be in a location most convenient for them
15. Presence of non-participants	Was anyone else present besides the participants and researchers?	Only the researchers were present
16. Description of sample	What are the important characteristics of the sample? (e.g. demographic data, date)	Demographics of the included participants are presented in the supplementary materials
**Data collection**		
17. Interview guide	Were questions, prompts, guides provided by the authors? Was it pilot tested?	The interview topic guide included open questions about their motivation for participation, and perceptions and experience of the programme and its components. It was developed collectively by the research team and patient representatives, and amended iteratively based on the interviews
18. Repeat interviews	Were repeat interviews carried out? If yes, how many?	Repeat interviews were not part of this study design and so were not conducted

19. Audio/visual recording	Did the research use audio or visual recording to collect the data?	Interviews were audio-recorded
20. Field notes	Were field notes made during and/or after the interview or focus group?	No
21. Duration	What was the duration of the interviews or focus group?	Average duration of the interviews was 40 minutes
22. Data saturation	Was data saturation discussed?	Data saturation was not discussed
23. Transcripts returned	Were transcripts returned to participants for comment and/or correction?	Transcripts were not returned to participants
**Domain 3: analysis and findings**		
**Data analysis**		
24. Number of data coders	How many data coders coded the data?	Analysis was led by EM and included peer discussion with JS and CA
25. Description of the coding tree	Did authors provide a description of the coding tree?	No coding tree data is presented
26. Derivation of themes	Were themes identified in advance or derived from the data?	Themes and categories were derived from the data

27. Software	What software, if applicable, was used to manage the data?	NVivo version 12
28. Participant checking	Did participants provide feedback on the findings?	No
**Reporting**		
29. Quotations presented	Were participant quotations presented to illustrate the themes/findings? Was each quotation identified? (e.g. participant number)	Quotations have been presented throughout the results, with participant codes assigned to all participants and identified with each quotation
30. Data and findings consistent	Was there consistency between the data presented and the findings?	We hope that the study findings have been clearly presented with data to illustrate the categories developed.<br>All major categories, and diversity of experiences reflected in subcategories, are presented in the discussion with supporting data in the appendices.
31. Clarity of major themes	Were major themes clearly presented in the findings?	
32. Clarity of minor themes	Is there a description of diverse cases or discussion of minor themes?	
